# Pituitary Adenylate Cyclase-Activating Polypeptide: 30 Years in Research Spotlight and 600 Million Years in Service

**DOI:** 10.3390/jcm8091488

**Published:** 2019-09-18

**Authors:** Viktoria Denes, Peter Geck, Adrienn Mester, Robert Gabriel

**Affiliations:** 1 Department of Experimental Zoology and Neurobiology, University of Pécs, 7624 Pécs, Hungary; mesteradri@gmail.com (A.M.); gabriel@gamma.ttk.pte.hu (R.G.); 2 Department of Immunology, School of Medicine, Tufts University, Boston, MA 02111, USA; peter.geck@tufts.edu

**Keywords:** PACAP1-38, migraine, PTSD, ischemia, Alzheimer, inflammation, cancer

## Abstract

Emerging from the depths of evolution, pituitary adenylate cyclase-activating polypeptide (PACAP) and its receptors (i.e., PAC1, VPAC1, VPAC2) are present in multicellular organisms from Tunicates to humans and govern a remarkable number of physiological processes. Consequently, the clinical relevance of PACAP systems spans a multifaceted palette that includes more than 40 disorders. We aimed to present the versatility of PACAP1-38 actions with a focus on three aspects: (1) when PACAP1-38 could be a cause of a malfunction, (2) when PACAP1-38 could be the cure for a malfunction, and (3) when PACAP1-38 could either improve or impair biology. PACAP1-38 is implicated in the pathophysiology of migraine and post-traumatic stress disorder whereas an outstanding protective potential has been established in ischemia and in Alzheimer’s disease. Lastly, PACAP receptors could mediate opposing effects both in cancers and in inflammation. In the light of the above, the duration and concentrations of PACAP agents must be carefully set at any application to avoid unwanted consequences. An enormous amount of data accumulated since its discovery (1989) and the first clinical trials are dated in 2017. Thus in the field of PACAP research: “this is not the end, not even the beginning of the end, but maybe the end of the beginning.”

## 1. Introduction

Pituitary adenylate cyclase activating polypeptide (PACAP) has been in the spotlight of extensive basic and applied research since its discovery [1,2]. The career of PACAP peptides (PACAP1-38 and PACAP1-27) is reflected by the ca. 3500 publications to date and highlighted in four excellent research articles published in *Nature* [3,4,5,6].

Three major binding sites have been recognized to mediate PACAP1-38 effects: PAC1, VPAC1, and VPAC2 receptors. The VPAC receptors also bind a PACAP-related peptide, vasoactive intestinal peptide (VIP) with the same high affinity as PACAP [7]. In other aspects, however, both VPAC1 and VPAC2 are unique entities. Their coding sequences show only 55% homology [8]. Several pharmaceutical agonists and antagonists differentiate between the two receptors with specific binding characteristics [7,9] and their tissue distributions show specific differences [10]. The complexity of PACAP receptors is further increased by PAC1 receptor (PAC1-R) isoforms through alternative splicing at the transcript level. In vertebrates, 20 PAC1-R isoforms have been identified [11], primarily in cell lines in vitro, but a number of them have also been verified in vivo with development-related expression patterns [12,13]. The isoforms were shown to affect ligand-binding selectivities and signaling mechanisms, but, in contrast to the large number of isoforms, only a few signal pathways are utilized. Nevertheless, differential expression of PAC1-R isoforms contributes to the differences in action exerted by the peptides.

The PACAP peptides were discovered as neurohormones first. PACAP1-38 was later recognized as a transmitter and neurotrophic mediator. In addition to the nervous system, PACAP and its receptors have been described in almost each bracket of the mammalian organism including various organs (e.g., pancreas, cardiovascular system, testes, gastrointestinal tract, etc.) [14]. Not surprisingly, approximately 40 different pathological conditions have been reported where PACAP1-38 may have clinical relevance (Table 1).

In the present review, we intended to follow the evolution of the PACAP system to elucidate why this highly conserved peptide has been spotted at the roots of nearly all physiological processes. Furthermore, to dissect disparate actions of PACAP1-38, we highlighted the six most extensively studied disorders and organize them according to PACAP1-38 involvement in pathophysiology, in prevention/treatment, and in conditions where its role happens to be ambiguous.

## 2. Emerging from the Depths of Evolution: PACAP/Glucagon Family

Despite its ubiquitous roles and pleiotropic regulations in various tissues, PACAP1-38 was discovered surprisingly late, as recently as 1989 [1], partly because of its unique characteristics. One of them, its highly basic charge was also fundamental in its discovery. The peptide was plotted as a highly basic releasing hormone “different from any other known releasing hormones,” according to Arimura, its discoverer [15]. Due to its positive charge, the majority of PACAP molecules circulate in ceruloplasmin complex [16], which is a unique feature among other circulating peptide hormones. This is critical to understand its availability in serum, the typical radioimmunoassay (RIA) based publications do not specify the biologically available free peptide levels, which is expected to be significantly lower. The majority of RIA assays measured 5–20 fmol/mL (15–60 pg/mL) total PACAP levels in serum [17]. Unfortunately, the biologically-available free peptide levels are not reported, but estimated to be an order of magnitude lower (0.5–2 fmol/mL or 1.5-6 pg/mL). Other short peptides with characteristics similar to PACAP and also act on G-protein coupled receptors (GPCRs) (e.g., adrenocorticotropic hormone (ACTH), parathyroid hormone, glucagon, gastrin) are different in several aspects. They have clear target organs and/or perform systemic functions in homeostasis. Their total plasma levels are several folds higher (50–150 pg/mL) than PACAP (American College of Physicians, Normal Laboratory Values, www.acponline.org) [18] and are not observed to form ceruloplasmin complexes. The low free peptide levels and the apparent lack of a systemic function strongly suggest that PACAP1-38 is a unique hormone. Most probably its major function is a paracrine operator instead of a systemic "master regulator." In the words of its discoverer: “although PACAP was a hypophysiotropic peptide, it was dissimilar to all other hormones” [15].

Its long evolutionary history also argues that it represents an ancestral form of local regulation with powerful local effects and an array of outcomes. PACAP is a ligand for GPCRs, which themselves evolved even earlier, in paleozoic archaebacteria and early eukaryotes. The extant GPCRs in multicellular organisms trace their origin from unicellular amoeboid eukaryotes with more than 700 hundred million years of history where cyclic-AMP (cAMP) and its GPCR receptor served as a stress signal [19]. The PACAP receptor family (part of the Secretin family) arose from the Adhesion receptor branch that regulate close contact local interactions [20,21]. The ancestral features of the PACAP system, i.e., stress signaling, cAMP pathways, and close contact local regulation, are still parts of its repertoire and represent important clues for understanding its physiology [22,23,24,25,26,27]. 

**Figure 1:**
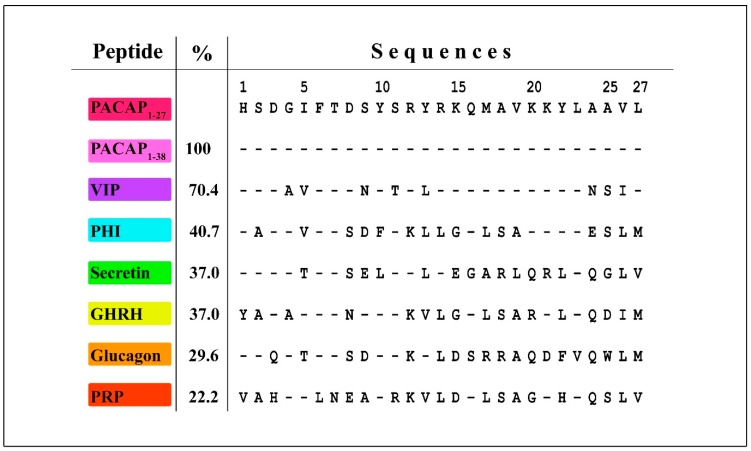
Core PACAP1-27 sequence homology within the human PACAP/glucagon family (PACAP paralogs). Peptide names and abbreviations are indicated at left. Amino acids are given in the single letter code, the numbers on the top show their positions. Sequence comparison was performed by BLASTP v.2.9.0 search on Non-redundant GenBank CDS translations + PDB + SwissProt + PIR + PRF databases. The numbers at right indicate percent homologies. Accession numbers: PACAP, (preproprotein) NP_001093203, VIP, AAB22264.1, PHI, 1010243A, Secretin, AAG31443.1, GHRH, AAH99727.1, Glucagon, NP_002045.1, PRP, (preproprotein) NP_001093203 [10].

The ligand for the PACAP GPCRs, the preproPACAP gene (ADCYAP1) evolved ca. 600 million years ago in Tunicates (Urochordates), which are the ancestors of all vertebrates [132]. PACAP emerged along with other vertebrate landmark features including the notochord and migratory neural crest cells, which further suggests a critical PACAP function in vertebrate biology [133]. Additional exon and gene duplications resulted in several paralogs in the PACAP/glucagon/secretin family that today are present on different chromosomes, but PACAP was the ancestral molecule [14]. GenBank searches of PACAP paralogs (see Figure 1) identified that the closest related hormone is the vasoactive intestinal peptide (VIP) with 19 identical residues compared to the 27 amino acid sequence of the short PACAP isoform, PACAP1-27 (70.4% homology). The other members include the peptide histidine isoleucineamide (PHI) with 11/27 identical residues (40.7% homology), both secretin and GHRH (growth hormone releasing hormone) with 37% homology, glucagon with 8/27 identity (29.6% homology), and PRP (PACAP Related Peptide) with 6/27 identical residues (22.2% homology) [134].

**Figure 2:**
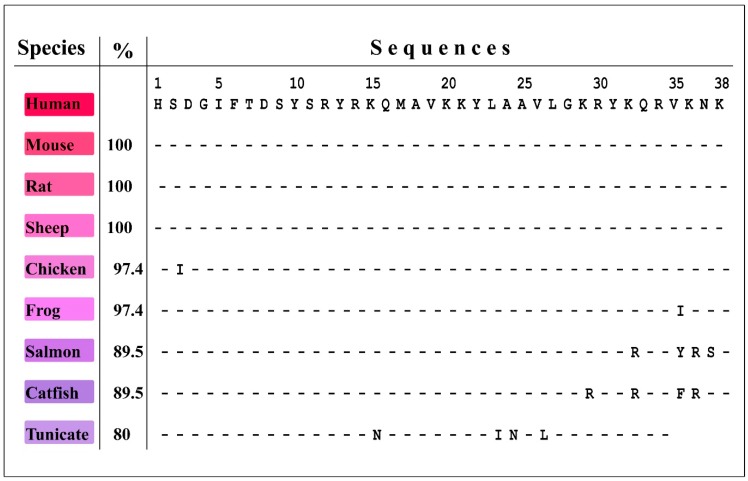
Comparison of species specific PACAP1-38 amino acid sequences (PACAP orthologs). The species names are indicated on the left. Percent homologies are calculated and shown in the “%” column on the left. Amino acids are given in the single letter code. The numbers on the top show their positions. Sequence comparison was performed by BLASTP v.2.9.0 search on Non-redundant GenBank CDS translations + PDB + SwissProt + PIR + PRF databases. Accession numbers: Human PACAP, (preproprotein) NP_001093203, Mouse, BAA28355, Rat, NP_058685, Sheep, AAB21469.1, Chicken, AAX56089.1, Frog (*Xenopus*), AAD56956.1, Salmon (*Oncorhynchus*), P41585.1, Catfish (*Ictalurus punctatus*), AAK66970.1, and Tunicate (*Chelyosoma productum*) [10].

In contrast, comparison of PACAP orthologs in various vertebrate species demonstrated an astonishing level of conservation, as shown in Figure 2. Not a single substitution occurred since mammalian branching ca. 80–100 million years ago. Only a single residue changed since the mammalian-reptile split ca. 250 million years ago (97% conservation) and only four positions were replaced since the urochordate-vertebrate separation ca. 560 million years ago (~80% conservation), which indicates enormous evolutionary pressure and a crucial PACAP function that has yet to be fully understood [10,134,135].

## 3. PACAP Receptors and Signal Transduction Bias

One of the surprising functional characteristics of PACAP is a wide spectrum of outcomes including potentially opposing effects [136]. This reported signaling bias is based on its complex pathways through multiple receptors, multiple receptor isoforms, and multiple signal transduction mechanisms. 

There are three major and several minor receptors that have been shown to bind PACAP isoforms and initiate a variety of intracellular signals. PAC1-R is a Type I receptor pharmacologically, with high affinity for PACAP, but low binding constant for VIP. Type IA receptors bind both PACAP1-27 and PACAP1-38, while Type IB receptors bind only PACAP1-38 with high affinity [137]. Although Type IB receptors have not been studied extensively, the 2018 IUPHAR/BPS database, a comprehensive database for pharmacological receptors [138], posits alternative splicing and G protein subunit/second messenger mechanisms in its specificity. The VPAC1-R and VPAC2-R receptors are Type II receptors with the same high affinities for both PACAP and VIP, comparable with the PAC1 receptor. PAC1-R was reported to display signal transduction bias through several signal pathways, while the Type II receptors use almost exclusively cAMP-PKA activation [14,139]. In addition, the secretin receptor also binds both PACAP and VIP with equal affinity and stimulates the same biological effect to the same extent as secretin, but the latter shows higher affinity [140].

The reported signal transduction bias of the PAC1-R receptor is governed by several splicing variations. The third intracellular domain (ICL3) can integrate three extra mini-exons (HIP, HOP1, and HOP2, a shorter variant of HOP1) in six splicing variations in rat (HIP, HOP1, HOP2, HIPHOP1, HIPHOP2, and the Null-variation) [5,141]. In humans, only HIP and HOP1 was detected with four splice variants [142]. This third variable intracellular domain is the binding site for a variety of G-protein α subunits. Depending on the splicing structure, different Gα isoforms can bind and initiate alternative signal cascades with bias for the adenylate cyclase-cAMP-PKA pathway or polyphosphoinositide-specific phospholipase C-dependent signaling. Another receptor variation in the transmembrane domain II and IV (PACAPR-TM4) activates an L-type calcium channel and calcium signaling [143]. Considering the lack of follow-up and mechanistic studies to explain the robust changes by a single point mutation, the validity of this variant needs further clarifications. In addition, PAC1-R activation can also lead to β-arrestin–mediated receptor internalization and endosomal signaling, which results in sustained Mitogen-activated protein kinase/MAPK/ERK pathway activation [144,145]. Furthermore, a VPAC1/RAMP2 (Receptor Activity Modifying Protein) complex in vitro shows phosphoinositide hydrolysis (unique in Type II receptors), with no change in cyclic AMP stimulation [146]. Other rare intramembrane, extracellular, and intracellular loop variations were also reported, which further increased the pharmacological and signal transduction complexities of PACAP receptors [147,148]. 

Altogether, more than twenty PACAP receptor variants have been identified in vertebrates [8]. Although these were detected in cell lines at first, several of them have also been verified in vivo. They show differential regulation in development indicating functional significance. [12,13]. The isoforms affect ligand-binding selectivities and may control the signaling bias. In contrast to the large number of receptors and variants, however, only a few signal pathways are activated. However, as the coreceptor composition and signal patterns may be tissue-specific representing tissue-specific transcriptional programs, a few signal pathways may still orchestrate tissue-specific responses. Nevertheless, differential expression of PAC1-R isoforms has been reported that probably contributes to the differences in action exerted by the peptides.

## 4. When PACAP1-38 Hurts

### 4.1. Involvement in a Migraine Attack

All primary headaches are considered severe neurological conditions causing poor quality of life and disability. Extensive investigations during the last two decades shifted the view of migraine pathophysiology from vascular mechanisms toward complex neuronal theories [149]. It has been suggested lately that migraine arises from inside the central nervous system and the vascular changes are only an epiphenomenon instead of the underlying mechanism. In our current view, a migraine could be considered as epilepsy of the sensory networks including the trigeminovascular system responsible for the throbbing headache. The central neural components that can trigger the dural-trigeminovascular reaction include multiple brain regions from the medulla up to cortical areas. The list in ascending order is astonishingly complex and includes the trigeminocervical complex, the rostro-ventral medulla, the dorsolateral pons, the peri-aqueductal grey matter, locus coeruleus, and superior salivatory nucleus. These areas are thought to be migraine generators that are modulated by diencephalic areas including hypothalamic as well as thalamic nuclei. Dural trigeminovascular activation, which is evidently causing the symptoms, is induced through the peripheral sphenopalatine or the trigeminal ganglions [149]. Ultimately, their fibers innervate the cranial vessels and release substances (i.e. PACAP1-38, VIP, calcitonin gene-related peptide (CGRP), Substance P, NO), which, in turn, evoke vasodilation, neurogenic inflammation, and mast cell degranulation [150,151,152].

A preponderance of evidence emerged in the last decade that PACAP1-38 is one of the potential inducers of migraine. Functional studies demonstrated that 10 pmol/kg/min PACAP1-38 infusion evoked headache and migraine-like attacks, in both healthy patients and migraineurs [102,103] and focused great attention on PACAP1-38. It is noteworthy that, since these pioneering studies, five clinical trials have been initiated using PACAP(s) as challenge agents to test drugs (e.g. Imigran, Sumatripan) in migraine patients. 

PACAP1-38 has been established in practically all aspects of trigeminovascular activation: (i) vasodilation of dural blood vessels [101,103,104], (ii) excitation of the central migraine generator [100], and (iii) mast cell degranulation [153]. One of the first actions attributed to PACAP1-38 after its discovery was a remarkable vasodilator effect [154,155,156]. The expression of all three PACAP1-38 receptors was reported in cranial/cerebral arteries in rats and humans [157,158,159]. The effect of blood-borne PACAP1-38 was a selective and long-term dilation of the external carotid artery and its branches (middle meningeal artery, superficial temporal artery) while the internal carotid artery and its middle cerebral branch remained unaffected. To date, the mechanism of the evoked vasodilation continues to be debated. Although circulating PACAP1-38 can pass the blood-brain barrier (BBB) to a certain extent via protein-transport systems (protein-transport system-6) [160], it is unlikely that PACAP1-38 could penetrate the walls of the above-mentioned big arteries to reach smooth muscle PAC1/VPAC2 receptors. Rather, it could be taken up via capillaries acting from inside out. Therefore, new considerations must be given to some central actions of PACAP1-38 toward inducing migraine. This is supported by the fact that all types of PACAP receptors were expressed by neurons of the trigeminocervical complex and the trigeminal and sphenopalatine ganglia [105,106,161]. Akerman [100] and colleagues’ elegant study showed that PACAP1-38 first evoked vasorelaxation of meningeal vessels through VPAC2 receptors, which is followed by an increase of spontaneous firing of trigeminocervical neurons after a 1.5-hour delay. Furthermore, the sensitivity of these neurons for intracranial dural stimulation also significantly increased. The delayed activation of the trigeminocervical complex was mediated through PAC1 as well as VPAC1-R. These landmark studies established a fundamental role of PACAP1-38 in the etiology of migraine.

PACAP1-38 is produced in the central nervous system in both neural and non-neural sources. PACAP1-38 expressing neurons have been identified in trigeminal and spheno-palatine ganglia that innervate extra-cranial and intracranial vascular structures in the head [157,162]. Furthermore, human mast cells were also reported to release PACAP1-38 [163]. Their role in migraine pathophysiology was corroborated earlier by studies reporting that histamine and prostaglandin I2 secreted by mast cells caused an immediate headache and migraine-like attacks [164]. Interestingly, in addition to PACAP1-38 expression, these special secretory immune cells also express PAC1 and VPAC1 receptors in a resting state and the VPAC2 receptor upon activation [165]. PACAP1-38 binding to its receptors causes degranulation of mast cells and induces an array of actions including skin edema or even dural vessel dilation [150,166,167]. The results showed that administration of PACAP6-38 (a potent mast cell degranulating agent through VPAC2 [168]) had no immediate effect on dural vessel diameter but exerted a delayed yet prolonged vasodilation. This result suggests that mast cells partially contribute to the vascular events prior to migraine, which implicates mast cells in the patho-mechanisms of the disease.

Mast cells, evidently involved in inflammation and an allergic reaction [169,170], also play a role in neurogenic inflammation. Sensory afferent nerve endings including those of the trigeminal nerve, release neuromodulators: calcitonin gene-related peptide (CGRP), substance P (SP), and PACAP1-38. Each of them has been investigated in both migraine and related neurogenic inflammation research, with mixed results [171]. CGRP did not seem to be involved in neurogenic inflammation and it induced neither mast cell degranulation in humans nor plasma protein extravasation [172,173]. Nevertheless, antagonists for CGRP (gepants) have been in the spotlight of pharmaceutical companies seeking migraine therapies. In contrast, SP has a strong degranulating effect on mast cells and is, thus, clearly involved in neurogenic inflammation. However, its failed SP receptor antagonist clinical trials for migraine [174] ruled out any roles in migraine pathophysiology. As discussed above, PACAP1-38 is implicated in neurogenic inflammation in the periphery and in migraine, but its role in dural neurogenic inflammation has yet to be investigated. Nevertheless, PACAP1-38 has a high impact on inflammatory processes in other tissues, which will be discussed later.

Considering the established negative effects of PACAP, systemic PACAP1-38 infusions may have side effects on the cardiovascular system. Involvement of PACAPs in the renin/angiotensin system and the presence of PACAP receptors in the coronary arteries [175,176] point to increased cardiovascular risk upon systemic PACAP treatment. While examining migraine patients, Birk et al. [102] reported that a severe increase in heart rate prevented the use of higher than 15 pmol/kg/min dosage of PACAP1-38 in humans. Seeliger and colleagues did not describe differences in heart rate or blood pressure upon 100 pmol/kg/h PACAP1-27 infusion but reported a significant increase of body temperature and persisting, six-hour long erythema suggesting that higher systemic doses should not be used in human in vivo experiments [177]. Unfortunately, their report did not present any data whether this low PACAP concentration induced a migraine attack or headache, and the Birk team did not mention any sign of skin edema or flushing either, which further underlined the need for standardized reporting criteria in the field. Furthermore, a clinical trial aimed at determining the maximum human dose would be essential and urgently needed. 

A current clinical trial (identifier: NCT03238781) [178] aims to evaluate the effect of a PAC1-R antibody in migraine patients. It is important to point out that, according our current knowledge, the migraine-inducing effect of PACAP1-38 is an inside-out action rather than a direct vasodilatory effect, and the passage of antibodies through the BBB is poor. Nevertheless, the antibody could block trigeminal PAC1 receptors since the trigeminal ganglion is located outside of the BBB. Therefore, a systemic antibody approach is feasible [179].

### 4.2. Contribution to Post-Traumatic Stress Disorder (PTSD) 

Although not life-threatening, PTSD is a severe, highly disabling psychiatric disorder and a large economic burden on the patients and on society at large. The symptoms include flashbacks, nightmares, severe anxiety, intrusive thoughts, arousal, and reactive symptoms that completely disrupt the daily lives of the afflicted.

Increased CRH (corticotropin releasing hormone) and ACTH levels in these patients prompted studies that proved the involvement of PACAP1-38 in the long-term stress-axis and a crucial role in the stress response [180]. PACAP1-38 was documented to play an essential role in the emergency response of the autonomic nervous system by stimulating catecholamine secretion from the adrenal medulla through sympatho-adrenal fibers [27,181]. The stress-response related brain regions (i.e., bed nucleus of stria terminalis—BNST, amygdala complex, hippocampus, and medial prefrontal cortex) were shown to express both PACAP1-38 and PAC1-R suggesting a role in stress, anxiety, and fear-related learning [182,183,184,185,186] through PACAP1-38-containing fibers that innervated CRH-expressing neurons in the paraventricular nucleus and BNST [187]. Second, PACAP1-38 was reported to upregulate CRH expression and production [188,189]. Third, although the *acute* stress response is PACAP1-38-independent, in the *sustained* stress response (with CRH release) PACAP1-38 signaling is essential [190]. To study anxiety levels, PACAP knockout mice were used. Through an initial screen for anxiety-related behavior, (i.e., open-field test, elevated plus maze, novel-object test) PACAP1-38 KO mice showed a lack of fear, hyperactivity, and increased exploration, which are related to reduced anxiety [191,192]. Another set of experiments aimed to investigate the effect of PACAP1-38 on psychotic behavior. Injecting PACAP1-38 into the paraventricular nucleus, central amygdala or bed nucleus of stria terminalis (BNST) resulted in significantly suppressed exploratory activities, and increased withdrawal coupled with immobility and enhanced startle behavior, respectively [186,187,193]. The findings point to PACAP1-38 contributions to anxiety-like responses through BNST circuits whereas its role in fear manifestation is linked to PAC1-R expression in the amygdala [194,195]. 

The processes of fear-learning, creating fear-memories and recalling fear have a tremendous impact on the development of an anxiety disorder. Extensive research of the last decade revealed more details about the function of the PACAP system with enhanced fear memories, which could lead to anxiety disorders like PTSD [196,197]. PACAP1-38 was reported to induce both short-term and long-term synaptic plasticity by regulating pre-synaptic and post-synaptic components [198]. For example, expression of activity-regulated cytoskeleton-associated protein (Arc/Arg3.1) improved memory performance in rodents [89,199]. In fact, PACAP1-38-induced memory formation also includes conditioned fear. PACAP1-38 exerts acute and chronic effects on the consolidation process of fear memory in the BNST, amygdala, or prefrontal cortex [199,200,201]. It is noteworthy that spatial memory performance was not affected by PACAP1-38 [200]. Mediation of these effects involves the PAC1-R receptor as an injection of PACAP6-38 or maxadilan into the BNST, which altered the stress-induced behavioral responses in cued fear conditioning [202]. Nonetheless, observations obtained on VPAC2 knockout mice suggest that this receptor might also be involved in fear memory formation. By testing Pavlovian fear conditioning, the VPAC2 knockout mice exhibited normal acquisition but aggravated extinction in terms of contextual and cued fear memory [203].

Apart from animal models, Ressler et al. [4] reported that a SNP, rs2267735 linked to the PAC1-R gene, predicted the development of PTSD in traumatized women. Interestingly, rs2267735 SNP maps the estrogen-response element of the ADCYAP1R1 gene, which indicates that PAC1-R expression is probably estrogen-dependent. Since this breakthrough study, this particular SNP has been confirmed to correlate with (i) increased amygdala reactivity, (ii) increased hippocampus reactivity, and (iii) reduced interaction between the two prominent brain regions [204]. A meta-analysis performed by Lindert et al. [205] also revealed that rs2267735 may increase the risk for PTSD.

Since the ancient PACAP system forms a bridge between the neuro-endocrine CRH/ACTH axis and brain areas in charge of emotional memory, it also links/mediates their pathologies. PACAP1-38 signaling through PAC1-R plays a key role in behavioral reactions to stress. Therefore, any imbalance of the signaling system could be responsible for the abnormal, sustained stress response called PTSD. 

## 5. When PACAP1-38 Rescues

### 5.1. Alzheimer’s Disease and PACAP1-38

Neurodegenerative diseases are a large and diverse group of incurable and debilitating conditions with progressive neuronal degeneration including Alzheimer’s disease, Parkinson’s disease, prion disease, Huntington’s disease, and spinocerebellar ataxia. Alzheimer’s disease is associated with dementia caused by slow and gradual death of nerve cells. At present, existing medications for Alzheimer’s diseases are very limited, and only treat the symptoms, rather than addressing the cause [206]. 

An early pioneering study reported that PACAP1-38 protects PC12 cells against β-amyloid-induced toxicity by suppressing the apoptotic machinery [207]. On cultured cortical neurons, Han et al. [83] reported that PACAP1-38 enhanced Sirtuin-3 production, which can protect stress-induced mitochondrial integrity and energy metabolism. PACAP peptides were also shown to increase the activity but not the expression of α-secretase enzymes in HEK 293 cells [208]. Amyloid precursor protein (APP) is processed by α-secretase. The cleavage results in the amyloid-β peptide and the release of the soluble N-terminal APP fragments (APPsα). Secretion of APPsα has proliferative, anti-apoptotic, and neuroprotective effects on neurons and prevents the deposition of β-amyloid peptide in plaques [209]. After intranasal administration of PACAP1-38 for three months in AD transgenic mice, APP processing shifted toward the non-amyloidogenic pathway and, in addition, brain-derived neurotrophic factor (BDNF) expression also increased as another beneficial effect of PACAP1-38 inhalation. Most importantly, the mice exhibited improved cognitive functions [84]. It would have been interesting to observe the anxiety levels of the animals (for explanation, see the PTSD section). Dogrukol-Ak et al. [210] achieved promising results by working out an alternative route to deliver PACAP1-38 into the central nervous system. They developed antisense compounds to selectively block the PTS-6 efflux pump that resulted in the accumulation of PACAP1-38 and 1-27. The increase in PACAP1-38 in such a way improved the learning ability of Alzheimer’s AD transgenic mice. They used the T-maze foot shock avoidance test to examine learning skills in this study. However, using a contextual fear memory paradigm is not an appropriate tool to study the association of PACAP1-38 with Alzheimer’s, even though the model is correct (for explanation, see the PTSD section).

In several studies, PACAP1-38 gene expression, protein levels, and the PAC1-R receptor were downregulated in both mouse models and postmortem human brain samples, which indicates correlations between PACAP1-38 levels and disease progression [82,83,211]. It seems that the decline of the PACAP system negatively correlates with plaque density and the severity of dementia. However, these results have provided only suggestions for the PACAP1-38 contribution to the pathogenesis. To clarify whether the malfunction of the PACAP/PAC1-R system is the reason or the consequence of Alzheimer’s disease, further experiments are warranted.

### 5.2. Ischemia/Stroke

Significant efforts of PACAP research are dedicated to investigate ischemic injuries. Ischemia is defined as an inadequate blood supply to a given tissue/organ due to blockage of blood vessels, which, in turn, leads to various levels of tissue destruction depending on the conditions. Local disturbance of the bloodstream can be caused by vasoconstriction, embolism, or thrombosis. Hypoxia is not the only challenge. The subsequent reperfusion-induced generation of reactive oxygen species (ROS) is an additional problem [212]. We will summarize potential PACAP1-38 therapy options for ischemic renal, cardiac, and hepatic injuries and review the results from two important areas including the brain and retinal ischemia.

PACAP1-38 has been shown to have a powerful vasodilator effect on cardiac vessels and the demonstrated PAC1-R expression in cardiac myocytes established the foundations for further studies to investigate the protective potential of PACAP1-38 in ischemic conditions [213,214]. A handful of publications showed that PACAP1-38 protected the cells in in vitro ischemia models [215,216] or in oxidative challenge [217]. A couple of papers have been addressed to the PACAP1-38 effect on hepatic ischemic damage as well. It has been established that all three PACAP receptors were upregulated during ischemia. Administration of PACAP1-38 in vivo unquestionably proved that PACAP1-38 ameliorated ischemic injury through multiple pathways: by inhibiting macrophage sequestration, suppressing pro-inflammatory cytokine release, and by attenuating apoptosis [218]. 

Investigations of PACAP knockout mice in cardiac, hepatic, or renal ischemia/reperfusion provided similar conclusions. The absence of endogenously produced PACAP1-38 resulted in increased vulnerability in ischemia and oxidative stress [219,220,221]. The results indicated a protective role for the endogenous PACAP system in pathophysiological conditions.

The following experiments provided a better understanding of the mechanisms in ischemic pathophysiology and the mechanisms of PACAP1-38 protection. In the heart, Alston et al. elucidated an interesting interaction between PACAP1-38 and locally-released cytokines. Elevated transcription of PACAP1-38 along with VIP in the heart as well as in the stellate ganglion after myocardial infarction was mediated by cytokine-activated gp130 signaling [219]. Those cytokines that activate gp130 (e.g., interleukin-6, corticotrophin-1) are considered as important survival factors for cardiac myocytes [222,223]. Therefore, it is reasonable to assume that the protective effect of the above-mentioned cytokines might be mediated by PACAP1-38. The attenuating effect of PACAP1-38 in the kidney is attributed to the suppression of Toll-like receptor 4/MyD88 signaling, known to be responsible for ischemia-induced inflammation. The protective effect was observed even if PACAP1-38 delivery was delayed by 24 hours [224,225,226]. Liu and colleagues [94] provided a new and curious insight by demonstrating that the protective effect of PACAP1-38 in the liver is accomplished through induction of the Yes-associated protein, which is an oncoprotein important in cell proliferation and regeneration. This protein is elevated in hepatocellular carcinoma [227]. This phenomenon (protecting the liver by inducing a cancer protein) is a fascinating example of the ambiguous functions of PACAP1-38 that will be discussed later. 

The ischemia-generated combined anoxia and hypoglycemia develop more severe conditions in neural tissues because the resulting apoptosis and oxidative stress are exacerbated by glutamate-induced excitotoxicity [228,229], with serious consequences [230,231]. Furthermore, neuronal tissues are much more susceptible to ischemic insults due to their poor regenerative capacities. Seeking therapeutic agents, therefore, is a priority in this field. In the present review, we give a general summary of ischemia in the central nervous system and discuss the details of retinal ischemic injury as a well-studied field within the central nervous system.

The retina is an outpost of the central nervous system, but it is exposed to higher risk of ischemia, not only by thrombosis or embolism but through other pathologies including diabetic retinopathy or glaucoma [232]. In both the bilateral common carotid artery occlusion model (BCCAO) and the intraocular hypertension ischemia/reperfusion model, dramatic cell loss was observed and reported. The morphometric analysis revealed that the total thickness of the BCCAO retina appeared to be half of the control retinas caused by severe cell death in the ganglion as well as inner nuclear layer three weeks post-operation [230,233]. Ganglion cell death was prevented, however, by either 10 fM or 10 pM intravitreally injected PACAP1-38, which indicated two distinct PACAP receptors with different affinities. The action of 10 fM PACAP1-38 was inhibited by a MAP kinase inhibitor, which suggested that the higher affinity PACAP receptor signaled through the MAPK/Erk pathway [234]. The results were confirmed by maxadilan, a PAC1-R agonist that exerted the same protective effect in BCCAO retinas, whereas the inhibitor PACAP6-38 prevented it [233,235]. Furthermore, Szabo and coworkers [236] showed that the underlying mechanism of PACAP1-38 protection markedly suppressed inflammatory cytokines, e.g., CNTF (ciliary neurotrophic factor), fractalkine, sICAM (soluble inter-cellular adhesion molecule-1), and IL-1 in the injured retina, similarly to other tissues. Moreover, PACAP1-38 treatment not only inhibits cell loss but enables the functionality of retinal circuitries [91]. An accurate and comprehensive metabolomics analysis characterizing the cell state and metabolic behavior of retinal tissue during the ischemic insult was conducted by D’Alessandro and colleagues [237]. Concomitant treatment with PACAP1-38 had strong and complex effects on the upside-down metabolic state induced by ischemia. First, PACAP1-38 exerted a multi-faceted blockade against glutamate-induced excitotoxicity by reducing glutamate, glutamine, and α-ketoglutarate levels. Second, it could fight oxidative stress, not only by decreasing pro-inflammatory factors, but also normalizing glutathione levels, which is an important free radical scavenger. Third, PACAP1-38 was able to redirect the metabolic flux from the pentose phosphate pathway toward glycolysis, which is a shift often observed in cells undergoing oxidative stress [238]. However, the peptide failed to restore energy metabolism and adenosine tri-phosphate (ATP) production [237]. Fourth, another alternative route for PACAP1-38 to cope with the ischemic challenge is to activate non-neural cell components in the retina, namely, microglia and macrophages. These cells produce anti-inflammatory interleukin-10, which may also contribute to the protective action of PACAP1-38 [239].

In addition to the above retinal ischemia observations, a result from studying brain ischemia further expanded our knowledge on PACAP1-38 functions: inhibition of caspase-3 activity [92] and inhibition of proinflammatory cytokine release [240,241]. Studies on microglia regulation by PACAP1-38 have revealed additional mechanisms in cerebral ischemia. First, PACAP1-38 seems to protect microglia in ischemia by inhibiting a microglial Toll-like receptor, MyD88 and by blocking the tumor necrosis factor α expression and NO production [240,241]. This finding might explain the increased cell number described in the retina by Wada et al. [239]. PACAP1-38 was shown to decrease proinflammatory mediators and, thereby, to resolve inflammatory processes, but also to induce the M2 phenotype transformation of microglia cells [90]. M2 is a preferred phenotype of microglia in neurodegenerative diseases since their presence is associated with regenerative growth, anti-inflammation, tissue repair, extracellular matrix reconstruction, or uptake of apoptotic cells [242]. This phenomenon resembles the role of PACAP1-38 in T-helper1/2 shift in peripheral inflammatory response that will be discussed in the next section. Furthermore, studying brain ischemia revealed that PACAP1-38 affects apurinic/apyrimidinic endonuclease 1/redox factor-1 (APE1) expression and function, which is an essential component of both DNA repair and redox signaling [243]. In addition, these studies also led to another important recognition. PACAP1-38 is a potent inducer of neural progenitors via the PAC1-R in the adult brain [244]. Other studies also support this observation. Under ischemia, the subventricular zone of the hippocampus contained significantly fewer proliferating progenitor cells in PACAP heterozygous knockout mice than in the normal control [95]. 

The role of VPAC receptors in cardiovascular side effects appears to be rather controversial. On the one hand, VPAC receptors could induce VEGF-mediated endothelial cell proliferation in vitro [245], while, on the other, VPAC2 receptors were reported to increase the severity of ischemic damage, hemorrhage, and, thus, the mortality of type-2 diabetic Goto-Kaizaki rats [246]. It is important to point out, however, that the diabetic condition was coupled with endothelial and vascular malfunctions [247]. Consequently, to elucidate the effects of VPAC2 receptor stimulation in ischemic injuries, this condition should be further investigated in non-diabetic models.

In addition to the direct effects mediated by PACAP receptors, PACAP also acts indirectly through the induction of neurotrophin release (e.g., BDNF), activation of trkB receptors, and attenuation of neuronal growth inhibitory signaling molecules p75NTR and the Nogo receptor [92,248].

In sum, PACAP1-38 could be an effective therapeutic agent for ischemic pathologies in both direct and indirect ways, and with both concomitant and delayed administration. The latter property could confer the peptide powerful potential in the recovery process following ischemic insults. The reported development of a promising new PACAP analog with higher resistance to dipeptidyl peptidase IV and, most importantly, with reduced cardiovascular effects in animal models offers a new avenue toward therapeutic applications [121].

## 6. When PACAP1-38 Appears to be Janus-Faced

### 6.1. Role of PACAP1-38 in Inflammation

There are two sets of available experimental data about the role of the PACAP system in inflammation, one confers on PACAP1-38 a broad range of anti-inflammatory effects and the other proves that PACAP1-38 is responsible for inflammation. According to the data comprehensively reviewed by Gomariz et al. [249], VIP/PACAP1-38 exerted purely anti-inflammatory effects on multiple sites of the mammalian immune system. Since the peptides are expressed in the same immune cell populations and target the same effectors, they are often referred to as the VIP-PACAP system sharing overlapping effects. The expression of their receptors, however, show differences. PAC1-Rs are borne exclusively by macrophages [250] whereas VPAC1-Rs are expressed by all immune cells (i.e., macrophages, lymphocytes, monocytes) [251,252,253]. The VPAC2 receptor was inducible in lymphocytes as well as in macrophages upon activation in rat [165], but its expression was constitutive by human lymphocytes [254]. Overall, both peptides regulate cytokine/chemokine production, predominantly by decreasing inflammatory factor expression [249]. They suppress mobility/migration by downregulating adhesion molecules and inhibiting infiltration of neutrophils [255]. Furthermore, VIP/PACAP1-38 control T-cell differentiation by pushing T-lymphocytes toward the T-helper2 fate [256], which is thought to foster anti-inflammatory signals [257]. By preventing T-helper1 cell differentiation and suppression of inflammatory interleukin production, PACAP1-38 treatment resulted in an improvement of multiple sclerosis symptoms [115]. 

Apart from studies where exogenously administered PACAP1-38 effects were examined, genetic modification of PACAP or PAC1-R genes also earned us valuable information about altered immune functions [258]. In PACAP knockout mice, airway lipopolysaccharide (LPS)-induced inflammation, dextran sodium sulfate-induced colitis, and multiple sclerosis were all markedly aggravated [117]. Likewise, PAC1 and VPAC2 knockout mice exhibited worsening symptoms of LPS-induced endotoxemia and delayed-type hypersensitivity, respectively [259,260]. The common motif observed in all of these immune diseases was enhanced inflammation. However, Jongsma and co-workers reported [261] that PAC1 knockout mice showed an immediate increased reaction to noxious stimuli (i.e., formalin) but appeared to be less sensitive later, which suggests that PAC1 signaling has a biphasic effect in pain transmission. More importantly, the observed decrease in the late phase response indicates that PACAP1-38 is involved in inflammation-induced nociception. 

It is important to point out that another set of reports presented evidence that the PACAP system contributes to inflammatory mechanisms. First, PACAP1-38 and its receptors are ubiquitously expressed in the neurons of dorsal root ganglia (sensory afferents), as well as in the spinal horn [262,263,264,265]. Elevated expression of PACAP1-38 in the sensory neurons in inflammatory circumstances implicated PACAP in neurogenic inflammation [64,266,267]. In neurogenic inflammation, sensory fibers release a large set of neuropeptides including calcitonin gene-related peptide (CGRP)/substance P (SP), PACAP1-38/VIP, and neuropeptide Y, which, in turn, cause various inflammatory responses (i.e., mast cells degranulation, vasodilation, plasma protein extravasation) [151,152,166]. The first functional report indicating that PACAP was a potent edema inducer was published by Warren and colleagues [77]. Both intradermal (10^−8^ M up to 10^−6^ M) and intravenous (7.5, 15, 30, and 100 pmol/kg/min) application of PACAP1-38 induced concentration-dependent skin edema and erythema in humans [177]. Significantly smaller edema was developed in PACAP knockout animals in neurogenic inflammation. Nevertheless, lacking PACAP1-38 did not influence the symptoms of non-neurogenic inflammation [76]. Investigation of ocular inflammation evoked by either electroconvulsive or endotoxin treatments resulted in the same conclusion. Neuropeptide release from the trigeminal sensory fibers caused the development of neurogenic inflammation. PACAP1-38 induced conjunctival hyperemia and protein extravasation was diminished by L-NAME, which is a nitric oxide synthase inhibitor. The finding demonstrates that NO not only mediates the effect of PACAP1-38 but, in turn, NO effectively induces neuropeptide release from nerve endings [69,268]. The results of a recent paper raise a novel possibility of applying PACAP1-38 eye drops in the dry eye syndrome, since the peptide potently increased lacrimation [269]. In retinal ischemic injury, external application of PACAP1-38 also seemed to be beneficial [99]. Although these reports did not indicate any signs of ocular inflammation upon treatments, reports of PACAP involvement in the pathogenesis of ocular inflammation warrant careful considerations. Chronic urinary inflammation was also accompanied by increased PACAP1-38 expression in both the dorsal root ganglia and the spinal cord. Moreover, both the immunoreactivity levels and the number of PACAP1-38 immunoreactive cells increased [64]. 

Altogether, PACAP1-38 appears to be involved in three types of inflammations: autoimmune-induced inflammation (e.g., rheumatoid arthritis, collagen-induced arthritis, Crohn’s disease), infection-induced inflammation, and neurogenic inflammation. In respect of the first two groups, PACAP1-38 has a remarkable attenuating effect, whereas it contributes to the pathophysiology of the latter.

### 6.2. Roles of PACAP in Stem Cell Regulation and Cancer Formation

Stem cells first evolved at the dawn of multicellularity where PACAP had important roles. Its adhesion receptor heritage and paracrine regulation appear to be central to maintain the stem cell niche. Stem/progenitor generating germinal zones of the brain are known as neurogenic niches including the subventricular zone of the lateral ventricles and the subgranular zone of the dentate gyrus. In this case, the extracellular matrix (ECM) and soluble factors maintain “stemness,” which involves self-renewal and multipotency [270,271]. PACAP was shown to upregulate ECM components and ECM-modifying enzymes in adult neural progenitors (aNPCs) that increased their surface adhesion in their niche [26]. These progenitors migrate in the rostral migratory stream (RMS) from the apical subventricular zone (SVZa) to the olfactory bulb in infant mice. PAC1-R co-localized with sites where progenitor adhesion was critical, which includes the progenitor niche (SVZa) and at their destination, but not in the migration process (in the olfactory tract) [272]. PACAP-regulated adhesion is important in stem cell homing as well. PACAP signaling was proved to induce bone marrow-derived stem cells to colonize into ischemic brains [273]. PACAP was also shown to promote neuroblast self-renewal [274] and to protect adult neural progenitors from stress-induced apoptosis [275]. This role, however, also has a Janus-face since experimental evidence demonstrated both pro-apoptotic and anti-apoptotic functions of PACAP1-38 in the postnatal mammalian retina [276].

Furthermore, PACAP1-38 promotes progenitor expansion by supporting proliferation. There are several reports that the PACAP/PAC1-R autocrine system is critical to extend the adult neural progenitor pool in the brain [244] and that appears to be the first step in astrocyte differentiation [277]. Not surprisingly, PACAP also has a role in retinal ontogenesis. It controls progenitor cell proliferation together with Kruppel-like factor 4 [278]. PACAP has a similar effect on olfactory neurogenesis. It promotes proliferation of the basal cell layer as well as the survival of the immature and mature neuronal layers [279]. Moreover, this role extends beyond the central nervous system. PACAP was reported to contribute to the proliferation of hematopoietic progenitor cells in murine bone marrow [280] and promotes proliferation of murine primordial germ cells as well [281]. 

PACAP also shows its Janus-face at the level of stem cell exit, by initiating stem/progenitor differentiation. PACAP was reported to both promote and block differentiation, depending on the stem cell niche and developmental stage. Astrocyte development, for instance, is induced from neural progenitors, but only after these cells have already generated neurons. This gliogenic switch is regulated by intrinsic and extrinsic factors and one of these positive differentiation factors is the PACAP/PAC1R system through the PACAP-cAMP-Ca(2+)-DREAM signaling cascade [282]. At different times during development, PACAP1-38 exerts different actions. It acts as an antimitotic factor on early neural progenitors directing neuronal differentiation, whereas, on late progenitors, it regulates the generation of oligodendrocytes [274,283]. Moreover, there is evidence that PACAP1-38 is also involved in the regulation of terminally differentiated astrocyte functions e.g., plasticity, glycogen synthesis, gliotransmitter production, etc. [284]. PACAP1-38 has been reported to promote survival, inhibit migration, and activate differentiation and neurite outgrowth of cerebellar granule cell precursors. In cerebellar neuroblasts, PACAP1-38 is a potent inhibitor of the mitochondrial apoptotic pathway [285]. The Lot1 transcription factor was reported as a critical mediator in the PACAP1-38/cyclic AMP differentiation pathway that negatively regulates cerebellar neuronal precursor proliferation [286]. Not surprisingly, in mice lacking pituitary adenylate cyclase-activating polypeptide, cerebellar development is seriously affected [287].

In contrast, PACAP1-38 counteracted hedgehog-dependent motor neuron production in mouse embryonic stem cells when the aNPCs were cultured in the absence of growth factors [288]. PACAP1-38 can also negatively regulate the proliferation of retinal progenitor cells through downregulation of cyclin D1 [289]. In the control of megakaryopoiesis and platelet production, PACAP1-38 and the vasoactive intestinal peptide (VIP) were reported to have a role through their common Gα-coupled receptor VPAC1. Treatment with PACAP1-38, VIP, or the adenylyl cyclase activator forskolin inhibits megakaryocyte differentiation, which results in thrombopathy and thrombocytopenia [290]. Injections with inhibitory anti-PACAP1-38 (PP1A4) or anti-VPAC1 (23A11) antibodies to block the pathway increased platelet numbers. The observation raised the possibility of clinical intervention for platelet recovery after myelosuppressive therapy [290].

Although modern cancer biology considers cancer as a Darwinian and adaptive tissue ecosystem [291], several lines of evidence indicate that most cancers are initiated and maintained by a single or very low number of cancer stem cells [292,293]. Mechanisms of stem cell regulation and dysregulation, therefore, are critical to understand cancer biology. The findings that PACAP1-38 regulates almost every aspect of stem cell physiology suggest that the PACAP1-38 system also contributes to cancer mechanisms. It is not surprising, therefore, that, in a lot of cancers of various pathologies, PACAP1-38 and its receptors are widely expressed and play opposing roles from activation to inhibition. 

Studying PACAP and PACAP receptor mechanisms in cancer is in its infancy. In most cases, we can only describe some fundamental observations, in a proof of principle manner. Most cancer cells have greatly dysregulated transcriptional programs and, consequently, many peptides and receptors are atypically expressed. 

However, it is not biologically insignificant. We have ample evidence that atypically expressed receptors for a growth factor are the very mechanisms of cancer initiation (e.g., Insulin-like growth factor receptor isoforms in lung cancer and HER2/neu in breast cancer). PACAP receptor overexpression in a stimulated environment with high neuroendocrine input may initiate dedifferentiation and cancer. For instance, prostate has a high neuroendocrine component and neuroendocrine prostate cancers are the most malignant. High PACAP or PACAP receptor levels in blood or urine may serve as future cancer markers.

In vitro studies detected PAC1-R binding sites in a number of cell lines from neural or neuroendocrine tumors. They were found in the human neuroblastoma NB-OK cell line [294,295], oligodendroglioma [296], and the neuroendocrine BON cells [297]. They were also present in non-neural cancer cell lines, like the medullary carcinoma 6/23 cell lines [298] and the rat pancreatic acinar AR4–2J cell line [299]. The Type II binding VPAC2-R gene was expressed in the hypothalamic GnRH neural cell line GT1–7 [300]. PACAP1-38 receptors have also been detected in adrenal pheochromocytoma PC12 cells [301] and adrenocortical NCI-H295 cells [302]. VPAC1-R mRNA was present in breast and intestinal cell lines, while both Type II receptors were expressed in neuroectodermal and pancreatic cell lines [28,303,304,305]. 

Apart from cultured in vitro systems, a long list of neoplastic tissues also expresses the receptors in vivo. Cancers from the lung [306] and colon [31] were positive for PACAP1-38 receptors. In particular, VPAC1-R was expressed in the mucosa and myenteric neurons. VPAC2 was detected in blood vessels, smooth muscles, and neuroendocrine cells. PAC1-R was found in myenteric neurons [34]. Type I receptors were found in breast cancer [307] and prostate cancer [46,308]. On the other hand, VPAC receptors were found in neural cancers, in human pituitary adenoma [309], and in brain glioma [310]. A large-scale study of human cancer tissues established that almost all human cancers express some forms of PACAP1-38 receptors and usually at higher levels than in normal tissues. Breast cancers (100% receptor incidence), prostate (100%), pancreas (65%), lung (58%), colon (96%), stomach (54%), liver (49%), urinary bladder (100%) carcinomas, lymphomas (58%), and meningiomas (100%) typically express the VPAC1-R. Leiomyomas express the VPAC2 receptor, whereas most paragangliomas, pheochromocytomas, and endometrial carcinomas express the PAC1-R [311].

The expression of PACAP itself has also been reported in human cancers including several neurological tumors. PACAP1-38 mRNA is expressed in most gliomas but only in 20% of meningiomas [296]. Significant PACAP1-38 expression was detected in human neuroblastomas [312,313], which suggests that it could also control neuroblastoma tumor cell proliferation. Most pituitary tumors show high PACAP1-38 expression [314]. Considering that cAMP is a mitogen in pituitary cells, PACAP1-38 may contribute to tumorigenesis [315]. In non-neurological tumors, PACAP1-38 is highly expressed in breast carcinoma [316] and in pancreatic carcinoma and pheochromocytoma [317]. PACAP1-38 has also been reported in ovarian tumors [318], prostate cancer cell lines [319], and cutaneous (Merkel) carcinoma [320]. 

The ubiquitous expression of the PACAP system in cancers strongly suggests that PACAP1-38 may play a role in the process. In fact, PACAP1-38 is showing its double face again with its capacity to both contribute and suppress malignancy. Astrocytomas show high expression of PACAP1-38 and its receptors. By selecting cell lines with increasing malignancy, PACAP1-38 levels did not change, but PAC1-R and VPAC1-R mRNA levels correlated with malignancy. Treatment by PACAP1-38 (10 pM) for three days resulted in increased proliferation that also correlated with malignancy. The results suggest that PACAP1-38 receptor expression and activity contribute to the malignancy and proliferative potential of astrocytomas [321]. PACAP1-38-27 stimulated colony formation in NCI-H838 cells, whereas the PAC1-R antagonist, PACAP6-38, reduced it, which indicates that PACAP1-38 promotes malignancy [322]. VPAC1 overexpression was linked with poor differentiation of colon cancer and epidermal growth factor receptor activation in cancer cells. In addition, VPAC1 overexpression in both blood vessels and macrophages in the tumor may also contribute to aggressive cancer [32]. An evolutionary stress-related effect of PACAP1-38, apoptosis protection also contributes to malignancy-promotion. Malignant peripheral nerve sheath tumors (MPNSTs) are sarcomas triggered by metabolic stress. Endogenous PACAP1-38-mediated signaling was reported to increase MPNST cell resistance to apoptosis from serum starvation and enhanced malignancy [323]. 

However, another set of reports demonstrated that PACAP1-38 actually suppresses malignancy. In the human glioblastoma cell line T98G, only the VPAC2 receptor subclass was expressed. VIP, PACAP1-38-27, and PACAP1-38-38 were potent and efficient inhibitors of cell proliferation, which established that PACAP1-38 is a tumor suppressor in this scenario [324]. The VIP peptide was also shown to inhibit human renal cell carcinoma proliferation [47]. Multiple myeloma is a plasma cell tumor in the bone marrow. PACAP1-38 suppressed myeloma-stimulated interleukin 6 (IL-6) secretion by the bone marrow stromal cells. By modifying the bone marrow milieu, PACAP1-38 suppressed myeloma cell growth indirectly [325]. By investigating the effect of PACAP1-38 on human retinoblastoma Y79 cell viability, 1–5 μM potently reduced retinoblastoma cell survival in a dose-dependent manner. In cognate peptidergic GPCR signaling, a 5-uM binding constant is typically not considered specific. The authors also ruled out PAC1 and VPAC receptors as mediators, which suggests novel PACAP1-38 pathways [48]. The significance of the finding is that, in paracrine operations, the local PACAP concentrations can be even higher. Therefore, it is not immaterial what signaling is active under these conditions. Horizontal cells are the cell-of-origin for retinoblastoma, which is a tumorous fatal childhood disease [326]. We found that intravitreal PACAP1-38 administration caused upregulation in PCNA protein levels in a stage-dependent manner and led to an increase in horizontal cells numbers, presumably via PAC1-R [49]. Our unpublished data shows that, prior to PCNA upregulation, PACAP1-38 downregulates Rb protein transcription [327]. The results clearly indicate that PACAP1-38 positively regulates horizontal cell numbers and postulate a novel possibility that PACAP signaling is critical in retinoblastoma.

Altogether, the data demonstrated the ubiquitous presence and activities of the PACAP system in both stem cells and cancer. PACAP was shown to affect every aspect of stem cell biology. It protects stem cells and promotes self-renewal not only in the neurogenic niche but in other niches as well. It promotes progenitor expansion by supporting stem/progenitor proliferation, but in the stem cell exit and differentiation, PACAP shows its double nature. Depending on the stage and tissue context, it can both promote and block differentiation.

The above functions and dysfunctions in stem cell regulation may be the link that associates PACAP to cancer. PACAP and its receptors appear to be expressed in various combinations in almost every cancer investigated, both in vitro in cell lines and in vivo in the cancer tissues. As far as its functions and biological effects are concerned, the confounding data point to both tumor suppression and tumor promotion. 

This almost universal ambiguity of the PACAP system may relate to a fundamental feature of this regulation, which is an ancestral stress response with short-term local operations. For instance, most cells respond to damage by apoptosis. As we showed, PACAP protects from apoptosis in almost every context, but the outcomes can be radically different. Protecting damaged stem cells in the retina may heal and rejuvenate, but blocking apoptosis in damaged lung and colon progenitors may generate cancer. The data established the power and the versatility of the PACAP system, but in the field of PACAP research “this is not the end, not even the beginning of the end, but may be the end of the beginning.” 

## 7. Conclusions

The data presented in this review demonstrate the extensive efforts to establish the role of the PACAP system in human biology and to take advantage of these peptides in therapeutic applications. However, despite this level of attention, no large-scale diagnostic or clinical applications are under way. The majority of clinical trials target only a few conditions (i.e., nephrotic syndrome, migraine, rosacea, cluster headache, major depression) and apply PACAP as a challenge agent, investigate its role to initiate disease, or test its involvement. We know only one ongoing clinical trial that attempts to test systemic injections of anti-PAC1-R as a potential tool to treat a migraine (identifier: NCT03238781) [178]. 

The reason is one of the inherent features of PACAP that it acts through a wide array of actions, which are both positive and negative. The unpredictable severity and number of side effects in both the nervous system and the peripheral organs argue against systemic application [328]. With respect to neurological/neurodegenerative disorders, investigations should focus on local administration. Several methodological approaches have been published that deserve consideration. For instance, several experiments support the idea of intranasal application, which have been already proven to be effective in treating both Alzheimer’s and Huntigton’s mouse models [84,89]. Transplantation of PACAP secreting stem cells has been applied successfully and appeared to be beneficial in the ischemic brain [90]. Specific inhibition of the PTS-6 transporter and consequent blocking of PACAP efflux through the blood-brain barrier is another option for taking advantage of the neuroprotective actions of PACAP [210].

Regardless, the take home message of this review is a warning. The available data strongly suggest that at any applications of PACAP treatments, the duration and concentrations of PACAP agents must be carefully considered to avoid unwanted consequences such as a migraine-attack, anxiety disorder, or even tumor formation.

## Figures and Tables

**Table 1:** Summary of the most relevant PACAP-related disorders.

Disorder	Implication	PACAP/VIP Receptor	Outcome	Literature
**Cancer**	pathogenesis/therapeutic		for details see Section 6.2	
Breast cancer	pathogenesis	PAC1, VPAC1, VPAC2		[28]
Cervical cancer	therapeutic	PAC1		[29,30]
Colon cancer	pathogenesis/therapeutic	PAC1 (Hip), VPAC1		[31,32,33]
Gastric cancer	pathogenesis	PAC1, VPAC1		[34,35]
Gliomas	pathogenesis/therapeutic	PAC1, VPAC1, VPAC2		[36,37]
Lung caner	pathogenesis	PAC1, VPAC1, VPAC2		[38]
Malignant peripheral nerve sheath tumor (MPNST)	pathogenesis	not determined		[39]
Malignant pheochromocytomas	pathogenesis	PAC1, VPAC1		[40]
Medulloblastoma	therapeutic	PAC1		[41,42]
Pancreatic cancer	pathogenesis	PAC1, VPAC1		[43]
Pituitary adenomas				[44]
Prostate	pathogenesis	PAC1 (null), VPAC1		[45,46]
Renal cell carcinoma	therapeutic	VPAC1		[47]
Retinoblastoma	unclear	PAC1		[48,49]
**Diabetes (Type II, insulin resistance)**	therapeutic	PAC1, VPAC1, VPAC2	- induces insulin secretion, - PAC1 antagonist impairs glucose tolerance - PACAP overexpression induces beta cell proliferation	[50,51,52,53,54]
**Diabetic retinopathy**	therapeutic	PAC1, VPAC1, VPAC2	- increases Akt and ERK1/2 phosphorylation - reduces the activation of p38 mitogen-activated protein kinase - reduces the expression of IL-1β in diabetic animals - downregulates VEGF and VEGF receptors, inversely regulates HIFs: downregulating HIF-1α and HIF-2α while upregulating HIF-3α - restores both Bcl-2 and p53 mRNA and protein expression - maintains synapse integrity	[55,56,57,58,59,60]
**Diabetic nephropathy**	therapeutic	not determined	- downregulates of several cytokines including CINC-1, TIMP-1, LIX, MIG, s-ICAM	[61]
**Inflammation**	Therapeutic/pathogenesis		for details see Section 6.1	
Allergic airway inflammation	therapeutic	PAC1, VPAC1, VPAC2		[62]
Atherosclerosis	therapeutic	not determined		[63]
Chronic cystitis, urinary bladder inflammation	pathogenesis	PAC1, VPAC1, VPAC2		[64,65]
Endotoxin induced airway inflammation	therapeutic	PAC1, VPAC1, VPAC2		[66]
Ileitis	therapeutic	not determined		[67,68]
Ocular inflammation	pathogenesis	not determined		[69]
Osteoarthritis	therapeutic	not determined		[70,71]
Pancreatitis	pathogenesis	not determined	- enhances caerulein-induced pancreatitis via downregulation of RegIII-β - lack of endogenous PACAP ameliorates symptoms	[72,73,74]
Skin neurogenic inflammation	pathogenesis	PAC1		[75,76,77]
**Toxoplasmosis**	therapeutic	VPAC1, VPAC2		[78]
**Human immunodeficiency virus (HIV) infection**	therapeutic	PAC1, VPAC1, VPAC2	- increases macrophage resistance to HIV-1 replication - reduces macrophage production of HIV-1 - induces the synthesis of β-chemokines and IL-10 - inhibits NF-kB, and reduced Cyclin D1 levels	[79,80,81]
**Neurological/neurodegenerative disorders**	Therapeutic/pathogenesis			
Alzheimer’s diseases	therapeutic	PAC1	for details, see Section 5.1	[82,83,84]
Bipolar disorder	therapeutic	PAC1	- no association with SNPs of PACAP gene - regulates binding between DISC1 and DISC1-binding zinc-finger protein responsible for neurite outgrowth	[85,86]
Epilepsy	therapeutic	PAC1, VPAC1	- during seizure PACAP is secreted and exert neuroprotective effects by regulating microglial phenotype, microglial interleukin secretion - inhibits long-term depression and depotentiation	[87,88]
Huntington’s diseases	therapeutic	PAC1	- improves memory performance	[89]
Ischemia/stroke (cardiac, renal, hepatic, retinal, brain)	therapeutic	PAC1	for details, see Section 5.2	[90,91,92,93,94,95,96,97,98,99]
Migraine	pathogenesis	PAC1, VPAC1, VPAC2	for details, see Section 4.1	[100,101,102,103,104,105,106]
Major depression (MDD)	therapeutic	not determined	PACAP regulated DISC1 mutation is linked to major MDD - SNP3 (rs1893154) of the PACAP gene is significantly correlated with MDD	[107,108,109,110].
Multiple sclerosis	therapeutic	VPAC1, PAC1	- prevents Thelper1 cell differentiation and suppression of inflammatory interleukin production	[111,112,113,114,115,116,117,118,119]
Parkinson’s diseases	therapeutic		- potent preventive molecule against cell loss and autophagy - facilitates dopaminergic neurotransmission - effective PACAP analog was developed with reduced cardiovascular side effects	[120,121,122,123]
Post-traumatic stress disorder	pathogenesis	PAC1 (VPAC2)	for details, see Section 4.2	[4,124,125]
Schizophrenia	therapeutic	PAC1	- SNPs of PACAP or PAC1 gene are associated with schizophrenia - regulates binding between DISC1 and DBZ responsible for neurite outgrowth	[86,110,126,127]
**Traumatic injuries**				
Traumatic brain injury	therapeutic	not determined	- attenuates neural injury by increasing level of SOD-2 and GPx-1 - suppresses T-cell response - decreases inflammatory interleukin levels	[128,129]
Spinal cord injury	therapeutic	PAC1	- activates axon regeneration through CRMP-2 and activation of glial elements	[130,131]

Legends: ERK—extracellular signal-regulated kinases; IL—interleukin; VEGF—vascular endothelial growth factor; HIF—hypoxia-inducible factor; CINC-1—cytokine-induced neutrophil chemoattractant; TIMP-1—metallopeptidase inhibitor 1; MIG—monokine induced by gamma interferon; s-ICAM—soluble intercellular adhesion molecule 1; NF-kB—nuclear factor kappa-light-chain-enhancer of activated B cells; SNP—single nucleotide polymorphism; DISC1—Disrupted-in-Schizophrenia-1; SOD-2—superoxide dismutase-2; GPx-1—glutathione peroxidase-1; CRMP-2—collapsin response mediator protein-2.
